# Taxonomic study of the genus *Unkanodes* (Hemiptera, Fulgoroidea, Delphacidae) from Pakistan, with description of a new species

**DOI:** 10.3897/zookeys.995.48766

**Published:** 2020-11-18

**Authors:** Kamran Sohail, Hassan Naveed, Daozheng Qin, Yalin Zhang

**Affiliations:** 1 Key Laboratory of Plant Protection Resources and Pest Management of the Ministry of Education, Entomological Museum, Northwest A&F University, Yangling, Shaanxi 712100, China Northwest A&F University Yangling China; 2 College of Life Sciences, Leshan Normal University, Leshan, Sichuan 614004, China Leshan Normal University Leshan China

**Keywords:** Distribution, Fulgoromorpha, key, morphology, taxonomy

## Abstract

Unkanodes (Kwonianella) malamjabbensis**sp. nov.** (Hemiptera, Delphacidae) is described and illustrated and *U. latespinosa* (Dlabola, 1957) is newly recorded from Malamjabbah, Swat, Pakistan. These two species represent the first records of the genus *Unkanodes* Fennah, 1956 from Pakistan. A key to the world’s species of the genus *Unkanodes* is provided.

## Introduction

The planthopper family Delphacidae currently consists of 2217 species in 427 genera (Bourgoin 2020). Delphacids are small insects that can be easily distinguished by the presence of a large, movable spur (the “calcar”) at the apex of the hind tibiae (Bartlett 2014). Most delphacids are grass-feeders, although some feed on other monocots such as sedges and rushes, and some feed on dicots (Bartlett 2019). A number of species feed on economically important crops, such as sugarcane, maize and rice (Wilson and O’Brien 1987; Wilson et al. 1994). The plant order Poales accounts for more than 70% of their hosts, while all other plant orders provide only 3%. In the Poales group, the family Poaceae has the highest (52%) percentage of hosts, followed by Cyperaceae (16.5%), while the remaining families account for only 3% of host records (Bourgoin 2020).

The delphacid fauna of Pakistan has been poorly studied, with only ten species previously recorded from this country (Bourgoin 2020). This figure, consisting of about 0.4% of the world’s described species, likely largely underrepresents the actual diversity of delphicids in this country. The genus *Unkanodes* was established by Fennah (1956) with the type species *Unkanodes sapporona* (Matsumura, 1935) from Che-Kiang (Zhejiang Province of China). Currently, this genus comprises nine species (Bartlett 2019) occurring in Afghanistan, Alaska, Austria, China, Denmark, Estonia, Finland, Germany, Greece, Iran, Japan, Lithuania, Mongolia, Poland, Russia, South Korea, Sweden, Taiwan, Turkey, Ukraine, Yugoslavia, and the U.S.A. (Bourgoin 2020). The genus *Unkanodes* is economically important and its members are vectors of many diseases in rice and cereals and also the causative agents of hopper burn diseases. *Unkanodes albifascia* is responsible for transmission of NCMV (Northern cereal mosaic virus), RBSDV (Northern cereal mosaic virus) and stripe disease. *Unkanodes sapporona* is involved in the transmission of NCMV, RSV (Rice stripe tenuivirus), and RBSDV. *Unkanodes tanasijevici* is reported to be a vector of IMMV (Iranian Maize Mosaic Nucleorhabdovirus), MIMV (Maize Iranian Mosaic Virus), MRDV (Maize Rough Dwarf Fiji Virus), and RBSDV (Bartlett 2019).

In this study, U. (Unkanodes) latespinosa (Dlabola, 1957) is recorded for the first time from Pakistan and a new species U. (Kwonianella) malamjabbensis sp. nov., is described.

## Materials and methods

Specimens were collected from Pakistan and deposited at the Entomological Museum of Northwest A&F University (**NWAFU**) Yangling, Shaanxi, China. Morphological terminology follows Asche (1985), Wilson (2005) and Bartlett et al. (2014). The method for genitalia preparation and clearing follows Wilson and McPherson (1980) and Wilson (2005). Morphological characters were observed using the stereomicroscope Olympus SZX10. Measurements of characters are given in millimeters (mm). Photographs of the adults were taken using a Zeiss AxioCam ICc 5. Adobe Photoshop was used for labeling and plate composition of the obtained images.

## Taxonomy

### Family Delphacidae Leach, 1815

#### Subfamily Delphacin.ae Leach, 1815

**Tribe Delphacini Leach, 1815**

##### Genus Unkanodes

Taxon classificationAnimaliaHemipteraDelphacidae

Fennah, 1956

80D7472E-4184-5DE2-A8B9-CE6A7448409F

Unkanodes Fennah, 1956.
Unkanodes sapporona (Matsumura, 1935), comb. by Fennah 1956: 474.

###### Type species.

*Unkana sapporona* Matsumura, 1935: 131, by original designation.

###### Diagnosis.

Relatively slender, head slightly narrower than pronotum. Vertex longer than broad, its width at base not exceeding width of an eye, shallowly rounded at apical margin; carinae of vertex and frons distinct. Frons parallel-sided, about 2.0–2.5 times as long as wide, lateral margins parallel, narrowing upwards in apical 1/3; median carina of frons bifurcates near fastigium. Lateral carinae of pronotum diverging, vanishing before reaching posterior margin. Calcar with 10–20 well-developed teeth; apical tooth separate from the remaining teeth. Posterior margin of pygofer with a cut on the sides. Segment X (anal tube) with a pair of teeth or teeth absent. Styli flattened, diverging or more or less parallel beyond middle, with complex apices, zigzag-shaped bent and wide or narrowed and slanting outwards. Armature of diaphragm (bridge of pygofer) bearing a pair of teeth directed upwards or a projection with 2 apices. Aedeagus more or less straight, or bent ventrad, elbow-shaped, slightly asymmetrical due to location of gonopore and arrangement of teeth on aedeagal shaft (after Fennah 1956 and Anufriev and Emeljanov 1988).

### Checklist of species of the genus *Unkanodes* Fennah

**Subgenus Unkanodes (*Chilodelphax* Vilbaste, 1968)**

**Unkanodes (Chilodelphax) silvaticus** Vilbaste, 1968

*Unkanodes silvaticus* Vilbaste, 1968: 24.

*Chilodelphax silvaticus* (Vilbaste, 1968); comb. by Kwon 1982: 4.

Unkanodes (Chilodelphax) silvaticus Vilbaste, 1968; comb. by Anufriev and Emeljanov 1988: 409.

**Subgenus Unkanodes (*Unkanodes* Fennah, 1956)**

**Unkanodes (Unkanodes) excisa** (Melichar, 1898)

*Liburnia excisa* Melichar, 1898: 67.

*Delphax excisa* (Melichar, 1898); comb. by Puton 1899: 108.

*Liburnia elymi* Jensen-Haarup, 1917: 3; syn. by Jensen-Haarup 1920: 53.

*Delphacodes excisa* (Melichar, 1898); comb. by Metcalf 1943: 436.

*Elymodelphax excisa* (Melichar, 1898); comb. by Wagner 1963: 167.

*Unkanodes excisa* (Melichar, 1898); comb. by implication Dlabola 1965: 86.

**Unkanodes (Unkanodes) latespinosa** (Dlabola, 1957)

*Calligypona latespinosa* Dlabola, 1957.

*Unkanodes latespinosa* (Dlabola, 1957), comb. apparently by Dlabola 1964: 240 (see also Dlabola 1967: 53; Emeljanov 1977: 113).

**Unkanodes (Unkanodes) paramarginata** (Dlabola, 1961: 275)

**Unkanodes (Unkanodes) sapporona** (Matsumura, 1935)

*Unkana sapporona* Matsumura, 1935.

*Unkanodes sapporona* (Matsumura, 1935), comb. by Fennah 1956: 474.

**Unkanodes (Unkanodes) tanasijevici** (Dlabola, 1965)

*Elymodelphax tanasijevici* Dlabola, 1965.

*Calligypona zeravshanica* Dubovsky, 1967; syn. by Emeljanov 1982: 98.

*Ribautodelphax notabilis* Logvinenko, 1970

**Subgenus Unkanodes (*Kwonianella* Anufriev, 1988)**

**Unkanodes (Kwonianella) albifascia** (Matsumura, 1900: 268)

*Liburnia albifascia* Matsumura, 1900: 268.

*Delphax albifascia* (Matsumura, 1900); comb. by Oshanin, 1907: 330.

*Delphacodes albifascia* (Matsumura, 1900); comb. by Metcalf 1943: 400.

Unkanodes (Chilodelphax) albifascia (Matsumura, 1900); comb. by Vilbaste 1968: 26.

*Chilodelphax albifascia* (Matsumura, 1900); status by Kwon 1982: 4.

Unkanodes (Kwonianella) albifascia (Matsumura, 1900); comb. by Anufriev and Emeljanov 1988: 409.

**Unkanodes (Kwonianella) insularis** Anufriev, 1988

Unkanodes (Kwonianella) insularis Anufriev & Emeljanov, 1988: 409.

**Unkanodes (Kwonianella) sympaticus** Anufriev, 1988

Unkanodes (Kwonianella) sympaticus Anufriev & Emeljanov, 1988: 409.

### Key to subgenera and species of *Unkanodes* of the world

This key is modified from Anufriev and Emeljanov (1988). Bartlett and contributors (2017) treated *Ribautodelphax notabilis* Logvinenko, 1970 as a synonym of *Unkanodes tanasijevici* (Dlabola, 1965) based on Nast (1987). In the present key, characters mentioned for *U. tanasijevici* are from the description of Ding (2006). Unkanodes (Unkanodes) paramarginata is not included in the key due to limited literature.

**Table:** 

1	Armature of diaphragm with a pair of teeth slanting upwards or directed back; genital style with relatively wide apices; segment X (anal tube) with large widely-spaced processes; sub genus Unkanodes (Unkanodes)	**2**
–	Armature of diaphragm without teeth or with a tooth bifurcate at apex; genital style with narrow apices; segment X (anal tube) with or without such processes	**5**
2	Process of anal tube spaced more widely, weakly or strongly diverging; genital style with strong subapical lobe, apex wider	**3**
–	Processes of anal tube spaced less widely, more or less parallel; genital style with weak subapical lobe, apex narrower (Anufriev and Emeljanov 1988: fig. 310: 1–12)	**U. (Unkanodes) excisus**
3	Process of segment X widely spaced weakly diverging; apex of genital style relatively wider (Anufriev and Emeljanov 1988: fig. 310: 13–17)	**U. (Unkanodes) sapporona**
–	Process of segment X widely-spaced strongly diverging; apex of genital style comparatively less wider	**4**
4	Aedeagus elbow-shaped; process of segment X posteroventrally curved (Figs 6–11)	**U. (Unkanodes) latespinosa**
–	Aedeagus straight with a strong tooth on dorsal aspect, ventrally with a weak lobe below the tooth; segment X not curved (Ding 2006: fig. 338A–L)	**U. (Unkanodes) tanasijevici**
5	Dorsal and posterior margin of pygofer forming an obtuse angle in lateral view; segment X (anal tube) with large widely-spaced teeth; apical half of aedeagus straight; subgenus Chilodelphax (Anufriev and Emeljanov 1988: fig. 311: 1–15)	**U. (Chilodelphax) silvaticus**
–	Dorsal and posterior margin of pygofer forming an acute angle in lateral view; segment X (anal tube) without or with narrowly spaced teeth; apical half of aedeagus directed dorsad (Anufriev and Emeljanov 1988); subgenus Kwonianella	**6**
6	Process of pygofer bridge very short, directed downwards, sometimes bifurcate at apex; genital style comparatively short with wide subapical lobe	**7**
–	Process of pygofer bridge slightly long, bifurcated and directed backwards; genital style longer with narrow subapical lobe or short with wide subapical lobe	**8**
7	Aedeagus near bent with a pair of long teeth perpendicular to shaft, the length of aedeagus matches with thickness of shaft (Anufriev and Emeljanov 1988: fig. 312: 17–19)	**U. (Kwonianella) sympatricus **
–	Aedeagus near bent without long teeth, the length of which matches with thickness of the shaft (Anufriev and Emeljanov 1988: fig. 312: 1–11)	**U. (Kwonianella) albifascia**
8	Aedeagus narrowing abruptly in apical 1/3, with a lobe-like process on ventral aspect in lateral view (Fig. 21)	**U. (Kwonianella) malamjabbensis sp. nov.**
–	Aedeagus not narrowing abruptly in apical 1/3, without a lobe on ventral aspect in lateral view (Anufriev and Emeljanov 1988: fig. 312: 12–16)	**U. (Kwonianella) insularis**

#### Unkanodes (Unkanodes) latespinosa

Taxon classificationAnimaliaHemipteraDelphacidae

(Dlabola, 1957)

29BF1511-A74B-5FD8-AEA5-2EFF5467DCD1

Figs 1–11

##### Remarks.

Dlabola (1957) described this species based on specimens from Afghanistan and provides a detailed description. It can be distinguished from other species of *Unkanodes* by the large, widely-spaced processes of segment X (anal tube) and elbow-shaped aedeagus.

##### Material examined.

3♂♂ (brachypterous), 7♂♂ (macropterous) Malamjabbah, Swat-Khyber Pakhtunkhwa, Pakistan, 35°13'21.76"N, 72°25'32.93"E, 2993.39 m, 5 vii 2018, sweeping grasses, coll. Kamran Sohail. The area has a very diverse habitat for fruits and vegetables, and this species was collected in grasses near vegetable fields. This species is newly recorded for the fauna of Pakistan.

##### Distribution.

Previously recorded from Afghanistan, Iran, Mongolia, Turkey and Yugoslavia. In this study it is recorded from Swat, Khyber Pakhtunkhwa-Pakistan.

**Figures 1–11.**
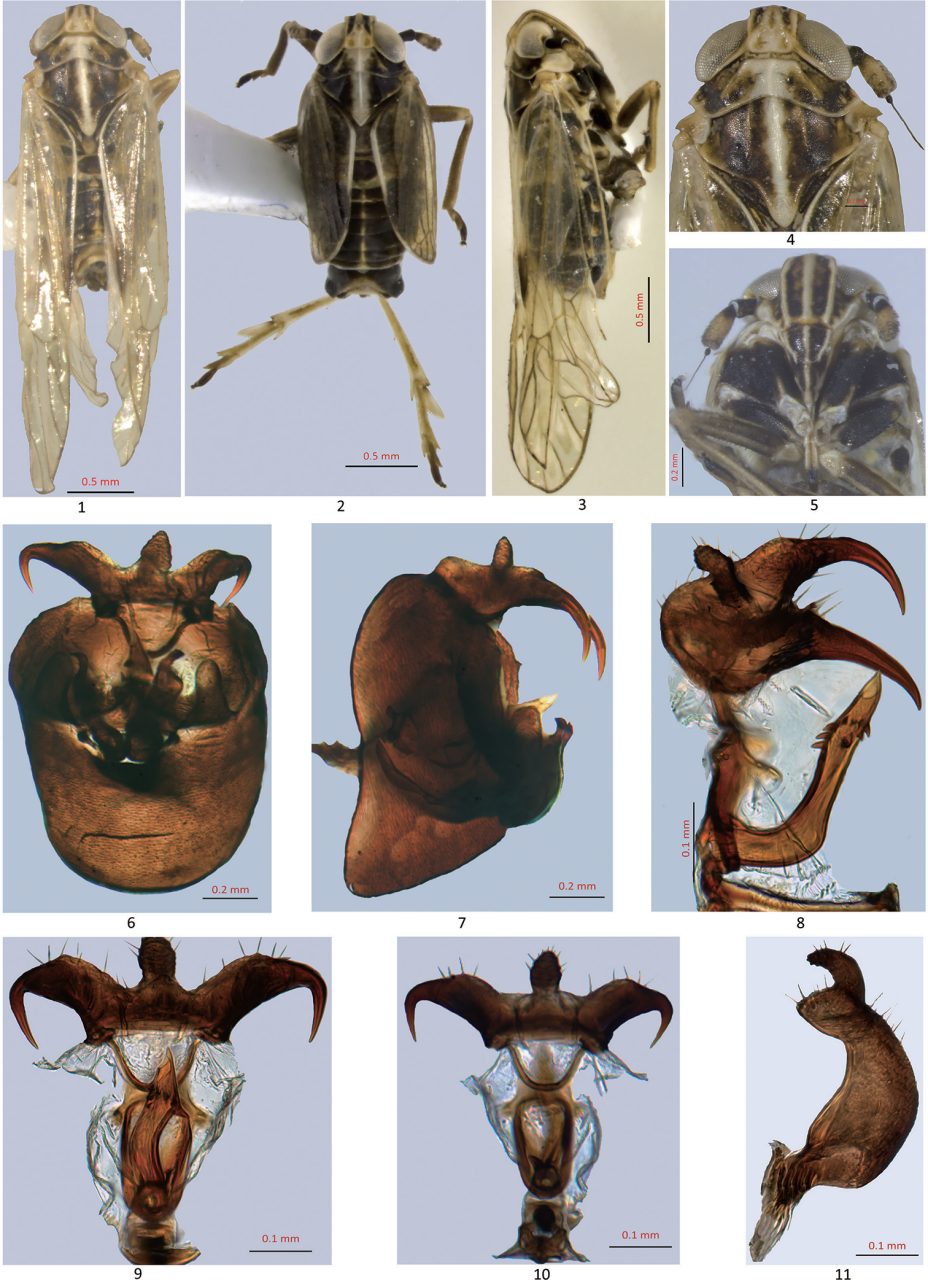
Unkanodes (Chilodelphax) latespinosa (from Pakistan) **1** adult, dorsal view (macropterous) **2** adult, same species (brachypterous) **3** adult (macropterous), lateral view **4** vertex, pronotum and mesonotum, dorsal view **5** frons, ventral view **6, 7** male genitalia, caudal and lateral views **8–10** anal segment and aedeagus, lateral, dorsal and ventral views **11** genital style, lateral view.

#### Unkanodes (Kwonianella) malamjabbensis sp. nov.

Taxon classificationAnimaliaHemipteraDelphacidae

0DE470A6-ECFA-5B5F-9ECF-8495EFDBEA29

http://zoobank.org/A9EFB5A5-AD3B-41B6-8599-94F3A4B3AC49

##### Description.

Length of male (n=2) 1.4–1.6 mm.

##### Colour.

General body colour dark brown to black. Vertex pale, compartments with three distinct yellow spots. Carina on frons pale, intercarinal region dark brown, gena concolourous with intercarinal region, compound eyes greyish. Antenna yellowish slightly darker at junction of scape and pedicel. Pronotum and mesonotum medially with a white stripe; darker at adjoining areas, extreme lateral margins and median carina white, lateral carina concolourous with adjoining regions. Forewings dark brown to black, apical and anal margins pale. Legs yellowish, spines with black apices. Abdominal tergites darker, segments IX and X lighter, pygofer brown.

##### Structure.

**Figures 12–17.**
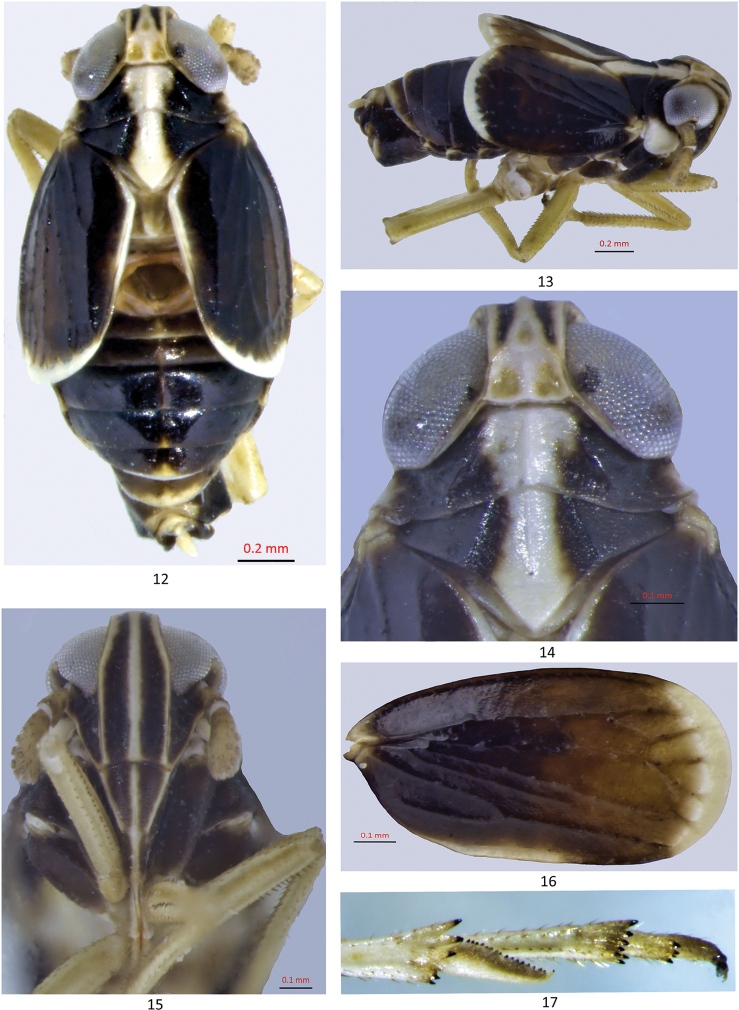
Unkanodes (Kwonianella) malamjabbensis sp. nov. **12, 13** adult, dorsal and lateral views **14** head and thorax, dorsal view **15** frons, ventral view **16** forewing **17** metatibial spur.

Head narrower than pronotum, eyes extending beyond posterior margin of vertex (Figs 12, 14). Vertex ca. 2X longer than wide; stem of Y-shaped carina of vertex obsolete, lateral and posterior margins distinct, arms of submedian carina meeting at fastigium (Fig. 14). Frons parallel-sided; widest near basal 1/4 of eyes, narrower in apical 1/3, median carina bifurcate near fastigium (Fig. 15). Antennal scape about as long as wide, ca 1/2 x length of pedicel, pedicel bearing many sensory pits arranged in longitudinal rows dorsally from base to apex (Figs 13, 15). Frontoclypeal suture distinct, slightly arched; median carina on postclypeus visible, rostrum elongate, reaching hind coxae (Fig. 15). Pronotum much wider than long at midlength; lateral carinae strongly diverging, vanishing before reaching posterior margin and not in line with mesonotal lateral carinae, anterior margin straight at vertex, posterior margin slightly concave medially (Fig. 14). Mesonotum tricarinate, subequal to length of pronotum; median carina not extending to apex of scutellum, lateral carinae slightly diverging reaching hind margin, tegula inconspicuous (Fig. 14). Forewing covering only half of abdomen; veins granulate (Figs 12, 16). Metatibiae with two lateral spines on shaft, first near tibiofemoral articulation, second after middle. Metatibial spur tectiform, distally narrowed bearing row of 18 black-tipped teeth on outer margin, inner margin straight (Fig. 17). Spinal formula of hind leg 5/7/4.

##### Male genitalia.

**Figures 18–25.**
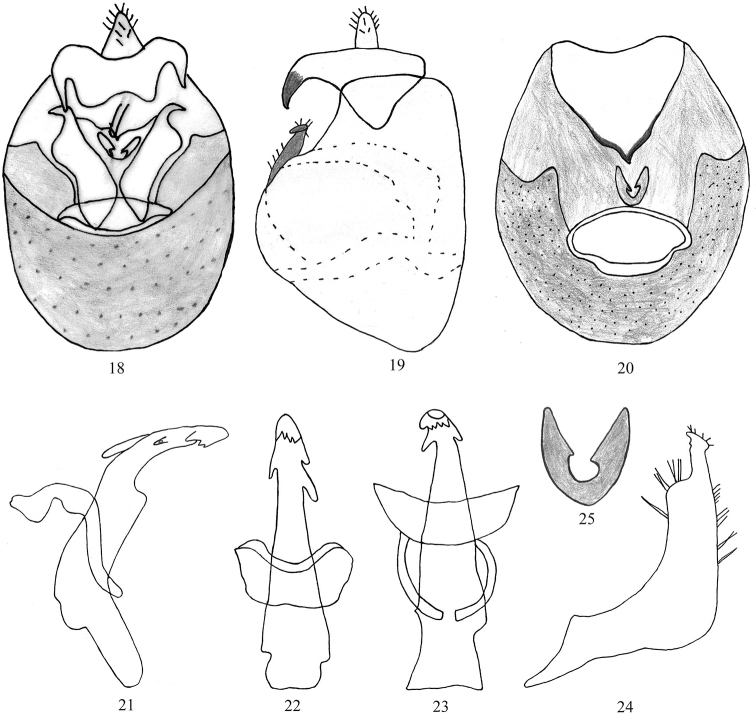
Unkanodes (Kwonianella) malamjabbensis sp. nov. **18, 19** male genitalia, caudal and lateral view **20** pygofer, caudal view **21** aedeagus, lateral view **22, 23** same, dorsal and ventral views **24** genital style, lateral view **25** medioventral process of armature of diaphragm.

In caudal view, pygofer wider than long widest at mid length, dorsolaterally nearly straight (Figs 18, 20); diaphragm armature well-developed, V-shaped, pair of lobes located near the parameres directed upwards, pygofer bridge bearings two distinct tooth-like processes widely diverging, directed backwards forming blunt apex (Fig. 20). In lateral view, anterior margin nearly straight gradually arched apically, dorsal and posterior margins acutely rounded (Fig. 19). Segment X (anal tube) bearing large, widely spaced posteroventrally curved acute processes (Figs 18, 19). Parameres longer than wide, apically narrow, subapical lobe wider, posterior margin straight (Fig. 24). Aedeagus elongate and narrow, basal 1/3 straight, bent gradually forming an obtuse angle, apical 1/3 gradually curved ventrad (Fig. 21); in lateral view, with lobe or hump-like process on ventral aspect, with a large tooth just above the lobe on the dorsal aspect (Fig. 21). Suspensorium angling circled laterally, apically wider (Figs 22, 23).

##### Type materials.

***Holotype*:** ♂ Malamjabbah, Swat-Khyber Pakhtunkhwa, Pakistan, 35°13'21.76"N, 72°25'32.93"E, 2993.39 m, 5 vii 2018, sweeping grasses, coll. Kamran Sohail. Paratype: 1♂, same data as holotype.

##### Remarks.

This new species was collected in a grass habitat near ponds. The Type locality is an understudied habitat for fulgoroids and the region reflects a true diversity of planthoppers for future prospects.

##### Female.

Unknown.

##### Etymology.

The new species is named after the type locality ‘Malamjabba’.

##### Diagnosis.

The new species is externally similar to U. (Kwonianella) albifascia which also has a white stripe on the thorax and median margins of the forewings. However, it can be separated by the distinctly separated process of the pygofer bridge, widely diverging in U. (Kwonianella) malamjabbensis sp. nov. but very short and bifurcate at the apex in U. (Kwonianella) albifascia (Anufriev and Emeljanov 1988, Figs 2, 6; p. 412); and apical half of aedeagus gradually curved ventrad bearing a lobe-like process on the ventral aspect in U. (Kwonianella) malamjabbensis sp. nov. versus the apical half of the aedeagus slanting dorsad without a lobe in U. (Kwonianella) albifascia (Anufriev and Emeljanov 1988, Fig. 4; pp. 409, 412). Unkanodes (Kwonianella) malamjabbensis sp. nov. is also close to U. (Kwonianella) insularis Anufriev and U. (Kwonianella) sympatricus Anufriev in external appearance but can be distinguished by the distinct shapes of the aedeagus and parameres.

**Figure 26.**
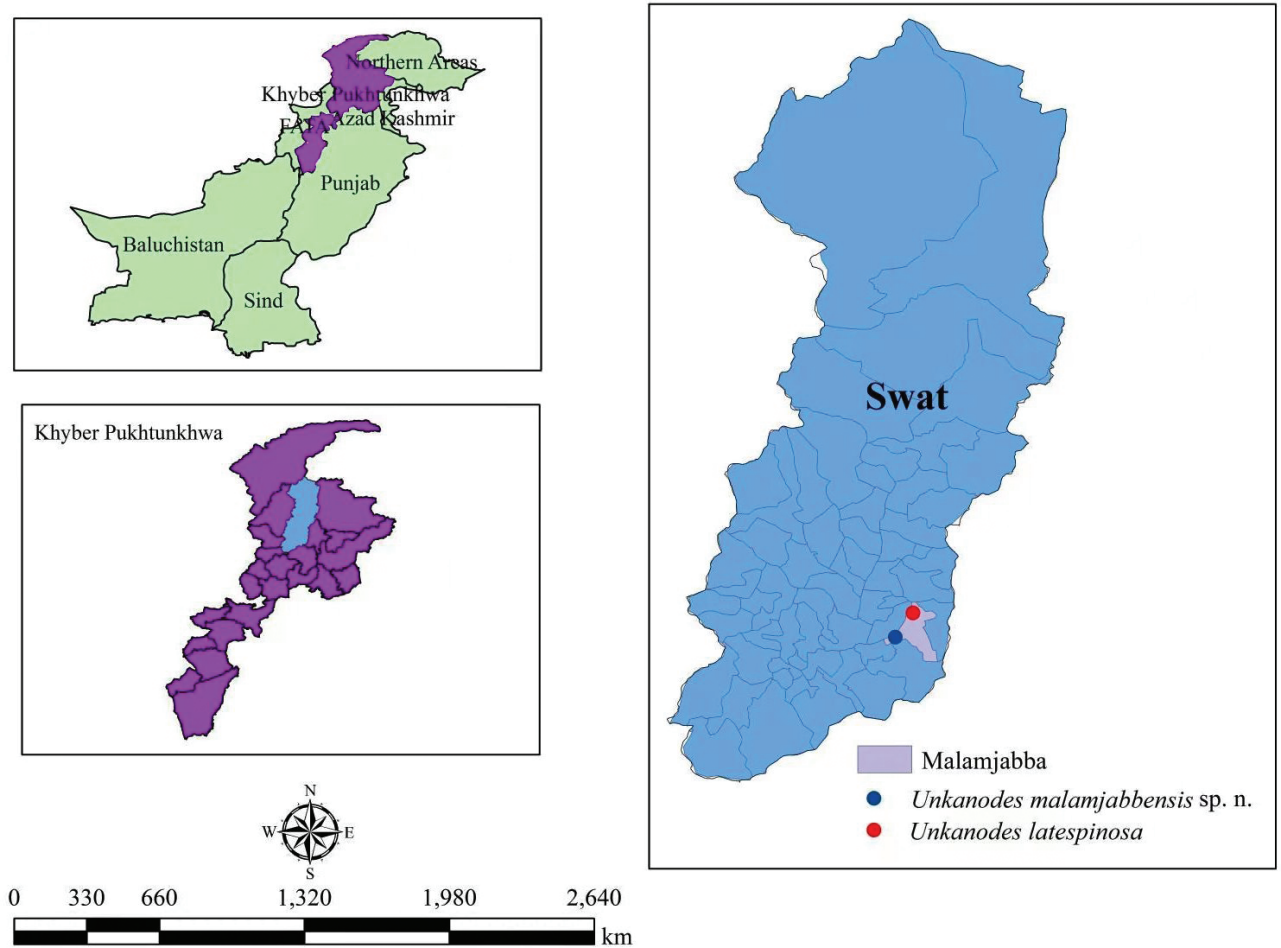
Distribution map of species of *Unkanodes* in Pakistan.

## Supplementary Material

XML Treatment for Unkanodes

XML Treatment for Unkanodes (Unkanodes) latespinosa

XML Treatment for Unkanodes (Kwonianella) malamjabbensis
